# Area of the cone interdigitation zone in healthy Chinese adults and its correlation with macular volume

**DOI:** 10.1186/s12886-018-0862-7

**Published:** 2018-08-01

**Authors:** Ruiping Gu, Guohua Deng, Yi Jiang, Chunhui Jiang, Gezhi Xu

**Affiliations:** 1 Department of Ophthalmology, Eye and ENT Hospital and Shanghai Key Laboratory of Visual Impairment and Restoration, Shanghai Medical College, Fudan University, Shanghai, 200031 China; 2 Department of Ophthalmology, The Third People’s Hospital of Changzhou, Changzhou, 213000 China; 3 Department of Ophthalmology, No. 5 People’s Hospital of Shanghai, Shanghai, 200240 China

**Keywords:** Optical coherence tomography (OCT), Cone interdigitation zone (IZ), Ellipsoid zone (EZ), The cone interdigitation zone area, Outer nuclear layer (ONL), Healthy Chinese adult

## Abstract

**Background:**

Numerous studies have suggested that the integrity of the cone interdigitation zone (IZ) could be considered to be a marker of photoreceptor damage and its recovery. However, little is known about the IZ in healthy eyes. Our present study was to measure the cone IZ area by optical coherence tomography (OCT), and determine its distribution in healthy adults.

**Methods:**

This was a cross-sectional non-interventional study. We involved a group of 158 emmetropic or low myopic (from −3D to + 0.5D) eyes in 97 healthy adult volunteers. All subjects underwent thorough ophthalmologic examinations and the posterior pole was scanned by OCT. The cone IZ area in healthy adults and its correlation with macular volume and other factors was analyzed.

**Results:**

The cone IZ was visible and clear in all 158 eyes, and the IZ area was successfully measured by 6 radical scans centered on the fovea. The mean IZ area was 30.22 ± 12.70 mm^2^, and ranged from 5.91 to 57.47 mm^2^. The IZ area exhibited a normal distribution (*P* = 0.635) with 95% confidence interval of 28.06–32.29 mm^2^. The IZ area was significantly correlated with the retinal and outer nuclear layer (ONL) volumes within the macula.

**Conclusions:**

The cone IZ area could be measured using a commercially available OCT system. The IZ area showed high variability among healthy adults, and this might be related to the variability in the photoreceptor distribution in healthy adults.

**Electronic supplementary material:**

The online version of this article (10.1186/s12886-018-0862-7) contains supplementary material, which is available to authorized users.

## Background

Optical coherence tomography (OCT), a noninvasive imaging technology, is able to provide detailed images and quantitative information about the retina’s structure and has become a standard diagnostic technique in ophthalmology [1–3]. With the spectral domain technique, the single highly reflective band at the outer retina that was observed using the original OCT devices [1–3] was resolved as three separate bands, corresponding to the photoreceptor Ellipsoid zone (EZ), the cone interdigitation zone (IZ), and the retinal pigment epithelium (RPE) -Bruch membrane (BM) compound [4]. The middle band, the IZ, represents the covering of the cone outer segments by apical processes of the RPE in a structure known as the contact cylinder [4–6]. Many studies have suggested that the integrity of the IZ could be considered to be a marker of photoreceptor damage and its recovery [7–11]. However, little is known about the IZ in healthy eyes. To date, only Rii et al. have examined this line, and reported that it was visible in 95% of healthy subjects [12]. Therefore; we performed the present study to improve our knowledge about the features of the IZ in healthy eyes, including the boundary of this line, its variability, and its correlation with other clinical factors.

## Methods

### Ethics

This study was approved by the Institutional Review Board of the Eye and ENT Hospital of Fudan University, and was performed in accordance with the principles of the Declaration of Helsinki. All of the subjects signed informed consent forms.

### Study participants

Health volunteers were recruited from the Department of Ophthalmology, Eye and ENT Hospital, Fudan University, between January 2016 and June 2016. All of the subjects signed an informed consent form. The study conformed to the tenets of the Declaration of Helsinki, and was approved by the Institutional Review Board of the Eye and ENT Hospital of Fudan University. All subjects underwent thorough ophthalmologic examinations, which included measurement of best-corrected visual acuity (BCVA), refraction measurement, intraocular pressure (IOP) measurement using a non-contact tonometer, measurement of axial lengths (AL) using an IOL master (IOLMaster500, Version 7.7, Carl Zeiss AG, Oberkochen, Germany), slit lamp microscopy, and an undilated fundus examination by direct ophthalmoscopy. The subjects’ medical and family histories were also collected. Inclusion criteria were as follows: BCVA ≥0.8, refractive index between − 3 diopters (D) and + 1 D, IOP < 21 mmHg, and AL < 25 mm. Exclusion criteria were prior history of ocular surgery or trauma, BCVA < 0.8, IOP ≥ 21 mmHg, AL ≥ 25 mm, presence of other ophthalmic abnormalities, family history of glaucoma in a first-degree relative, or a systemic disease that might have ocular involvement (e.g. diabetes mellitus or hypertension).

### OCT imaging

All OCT images were obtained by a spectral domain system (Spectralis, ver. 1.5.12.0; Heidelberg Engineering, Heidelberg, Germany) with a normal pupil. Two scanning procedures were used: (1) a radial scanning pattern with 6 scan lines centered on the fovea and covered a 30 degree round area (1042 A-scans per line, each line comprising 100 averaged scans obtained using eye tracking); (2) a posterior pole volume scan using 97 raster lines, each line comprising 30 averaged scans, covering an area of 30 × 25 °. Only scans with good signal strength (signal-to-noise ratio, ≥ 20 dB) were saved for analysis.

### Measurement of the cone IZ area

**Fig. 1:**
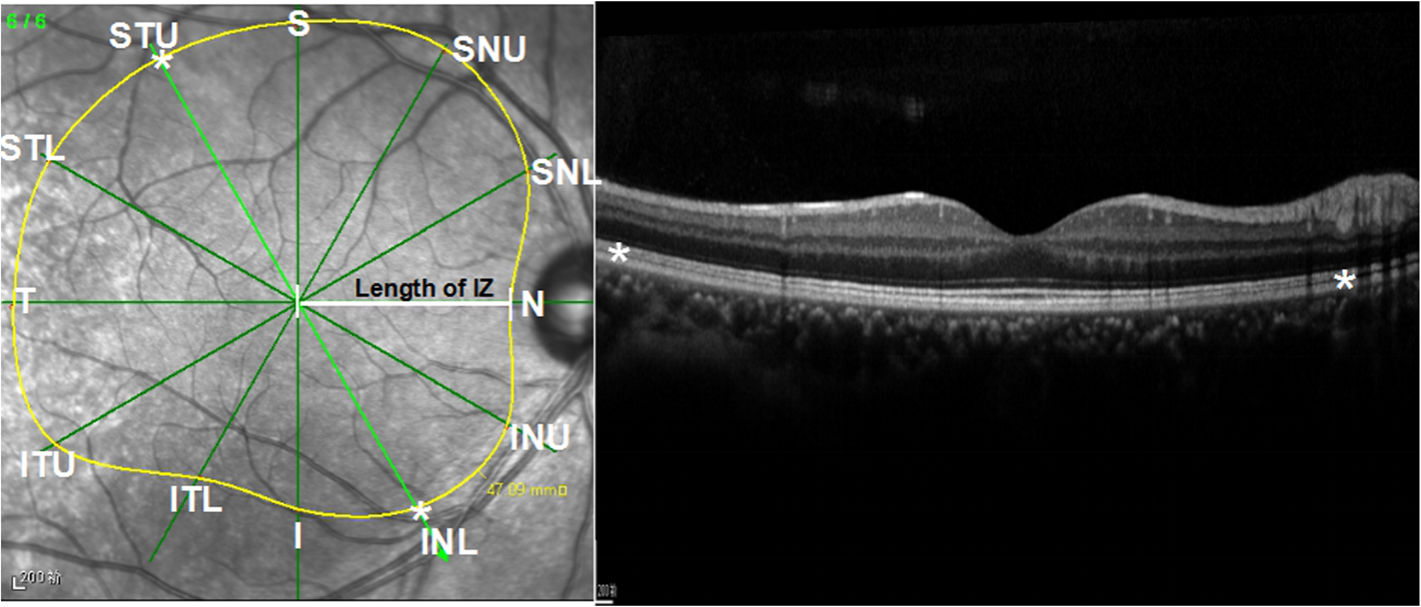
Measurement of the IZ area and IZ length in 12 directions. The boundary of IZ was defined as the ends (*) of the continuous and smooth, highly reflective line between the ellipsoid zone and the retinal pigment epithelium-Bruch membrane (RPE-BM) compound. The length of the IZ was measured in each direction from the central fovea to the end of the IZ. IZ = interdigitation zone; INL = inferior nasal lower; INU = inferior nasal upper; ITL = inferior temporal lower, ITU: inferior temporal upper, I: inferior, N: nasal, S = superior; SNL: superior nasal lower; SNU = superior nasal upper; STL = superior temporal lower; STU: superior temporal upper; T = temporal

Scans from the radial pattern were used to measure the IZ area. The boundaries of the IZ were defined as the ends of the continuous and smooth highly reflective line between the EZ and the RPE-BM compound on each of the 6 radical scans, with 12 markers for each eye. Then, the IZ area was automatically calculated by the built-in software. The length of the IZ (the distance between the center of the fovea and the boundary of IZ in all 12 directions) was measured manually (Fig. 1). The IZ was measured by one experienced ophthalmologist, who was masked to the subjects’ characteristics.

### Macular volumes

The results of the posterior pole volume scan were processed according to the macular thickness protocol [13]. The entire macula was divided into 9 regions with 3 concentric rings measuring 1, 3, and 6 mm in diameter centered on the fovea according to the Early Treatment Diabetic Retinopathy Study protocol [14]. The RPE-BM compound was defined as the region from its inner border to its outer border. The full retina was defined as the region from the inner internal limiting membrane (ILM) to the outer border of the RPE-BM compound. The outer nuclear layer (ONL) was defined as the region from the outer border of the outer plexiform layer (OPL) to the external limiting membrane (ELM). The ELM-BM was defined as the region from the ELM to the outer border of the RPE-BM compound. The volumes of these retinal layers within the foveal and para/perifoveal areas were automatically calculated using the built-in software. The photoreceptor layer volume was calculated as the ELM-BM volume minus the RPE-BM compound volume. The volumes of the different layers of the macula were calculated by adding the volumes of the specified layer in the fovea, parafovea, and perifovea.

### Repeatability and reproducibility

The first 20 eyes were included to test the repeatability and reproducibility of the method. Two series of radial patterns were taken by OCT in a single visit to determine the repeatability of the IZ area measurements. The intra-observer repeatability of the IZ area was evaluated by measuring the IZ area twice from the same set of radical scans. For inter-observer reproducibility, two observers each measured the same set of scans separately. Intraclass correlation (ICC) and Bland–Altman plots were used to assess the repeatability and reproducibility of the measurements.

### Statistical analysis

All analyses were performed using SPSS for Windows, ver. 17.0 (SPSS, Inc., Chicago, IL, USA). The IZ areas were plotted on a frequency histogram. The Kolmogorov–Smirnov test was used to assess the distribution of the IZ area. Analysis of covariance was used to test the correlation between the IZ area and the volumes of each retinal layer and other clinical variables. The differences in the IZ area and lengths in all 12 directions between the right and left eyes were tested using Student’s *t* test. The binocular symmetry of them was tested by paired *t* tests.

## Results

A total of 158 eyes in 97 healthy volunteers met the inclusion criteria, including 48 (49.48%) males and 49 (50.52%) females. The mean ± standard deviation age was 32.13 ± 12.08 years (range, 20–61 years), IOP was 14.41 ± 2.89 mmHg, axial length was 23.54 ± 0.79 mm, BCVA was 0.01 ± 0.05 (logarithms of the minimum angle of resolution), and the spherical equivalent was 0.80 ± 1.04 D (Additional file 1: Table S1).

### The cone IZ area

**Fig. 2:**
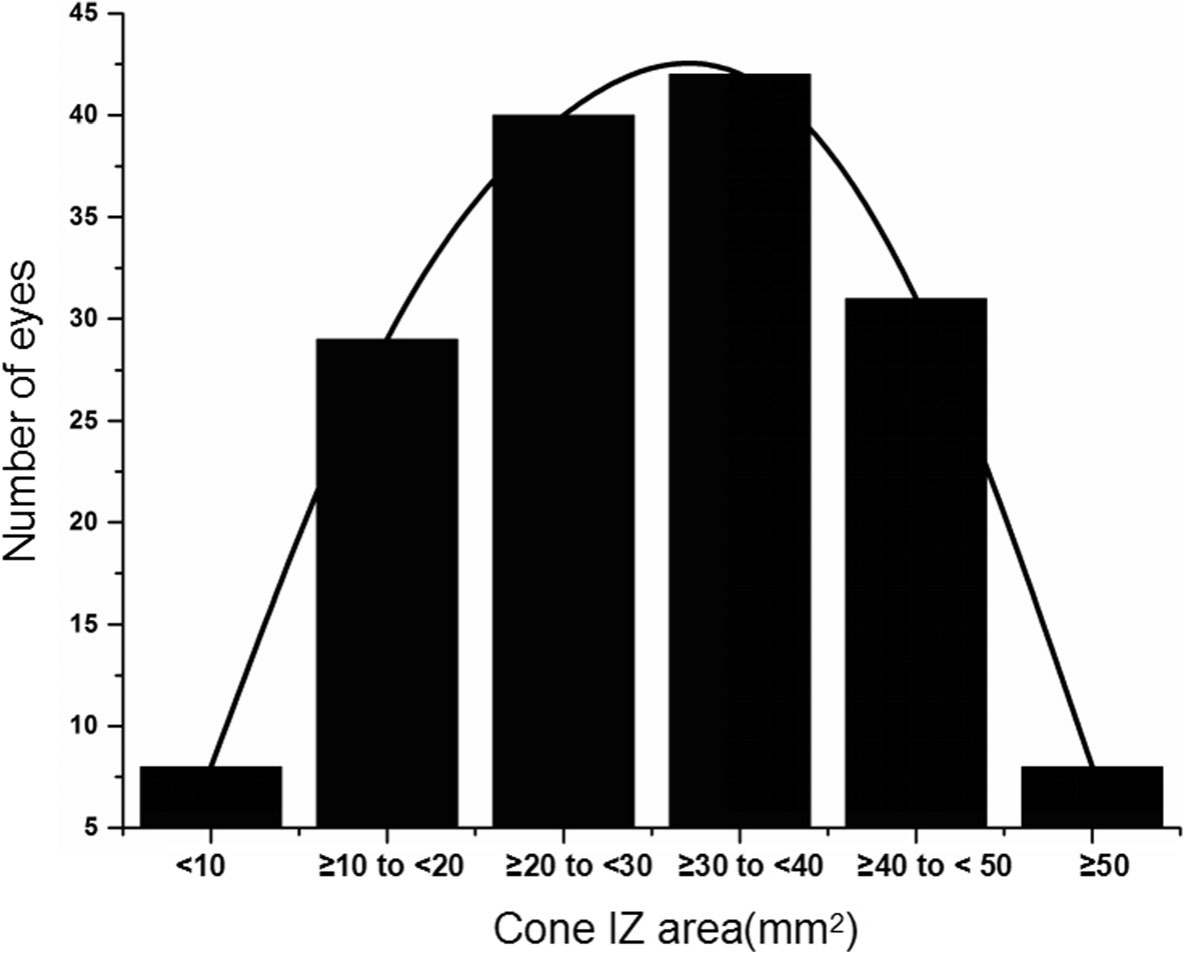
Frequency distribution of the cone IZ area in healthy adults. The Kolmogorov–Smirnov indicated that the IZ area exhibited a normal distribution (*P* = 0.635). IZ = interdigitation zone

The IZ was visible and clear in all the radial scans in all 158 eyes. The mean ± standard deviation IZ area was 30.22 ± 12.70 mm^2^ (range 5.91–57.47 mm^2^). Kolmogorov–Smirnov test indicated that the IZ area exhibited a normal distribution (*P* = 0.635) (Fig. 2) with a 95% confidence interval of 28.06–32.29 mm^2^. The IZ area and the IZ lengths in all 12 directions were similar in the left and right eyes (all *P* > 0.05) **(**Additional file 1: Table S2). The IZ area and the IZ lengths in most of the 12 directions showed good inter-ocular symmetry (Additional file 1: Figure S1, Table S3). And the cone IZ area showed good symmetry (29.74 ± 13.61 vs. 29.79 ± 12.39 mm^2^, *p* = 0.981) (Additional file 1: Table S3) and significant correlation on binoculus (*r* = 0.916, *p* <  0.0001).

### Correlation between the IZ area and other variables

The IZ area on OCT measurement was correlated with the volume of full retina (*r* = 0.274, *P* = 0.01), ONL (*r* = 0.351, *P* <  0.0001), as well as the volumes of ELM-BM (*r* = 0.324, *P* <  0.0001), and photoreceptor (*r* = 0.351, *P* <  0.0001) of the macula, but not with age, gender, SE, IOP, or AL (Table 1).

**Table 1:** Correlations between the interdigitation area and other clinical or OCT variables

	*r*	*P*
Sex	−0.068	0.393
Age (years)	0.006	0.94
AL (mm)	0.076	0.341
SE (D)	−0.158	0.148
IOP (mmHg)	0.006	0.94
BCVA (LogMAR)	0.075	0.351
Volume of the macular area		
Retina volume	0.265	0.01
ONL volume	0.384	< 0.0001
ELM-BM volume	0.324	< 0.0001
Photoreceptor volume	0.357	< 0.0001
RPE-BM volume	0.111	0.233

*AL* axial length, *BCVA* best-corrected visual acuity, *BM* Bruch membrane, *D* diopters, *ELM* external limiting membrane, *IOP* intraocular pressure, *LogMAR* logarithms of the minimum angle of resolution, *ONL* outer nuclear layer, *RPE* retinal pigment epithelium, *SE* spherical equivalent. Analysis of covariance was used to determine the correlations between the interdigitation area and the volumes of each retinal layer and other clinical variables

### Repeatability and reproducibility of the IZ area measurements

The mean ICCs for intra-observer repeatability and inter-observer reproducibility of the IZ area were 0.982 and 0.974, respectively. The mean ICC for the repeatability of the IZ area was 0.981. The Bland–Altman plots also showed good repeatability and reproducibility of the method (Additional file 1: Figure S2).

## Discussion

OCT was introduced into the field of ophthalmology many years ago [1]. More than 18 anatomic landmarks can be discerned by OCT [6], including the IZ. The IZ is a cylinder joining the RPE apical processes to the external portion of the cone outer segment [4, 5]. To our knowledge, our study was the first to measure the IZ area in a group of healthy adults. The repeatability and reproducibility tests showed good reliability of the measurement method. The mean IZ area was 30.22 ± 12.70 mm^2^ (range, 5.91–57.47 mm^2^). Although the IZ area showed a normal distribution, it was highly variable. The area was also closely correlated to the full retina and ONL volumes.

Considering the IZ area was successfully measured in all subjects with good repeatability and reproducibility, the methods used here could be reliable for evaluating the state of photoreceptors, mainly cones, in macular studies. The IZ could still be observed when using just one OCT scan line, but the two-dimensional area is probably more clinically relevant than the one-dimensional length. Xu et al. conducted a study of patients with a macular hole and found that the preoperative base area of the hole (*P* <  0.0001), but not the minimum diameter of the hole, was a predictor of postoperative BCVA [15]. Additionally, Oh et al. reported that the preoperative EZ defect area, but not the EZ defect diameter, was correlated with the visual improvement in patients with a macular hole [16]. Therefore, the IZ area should be more closely related to the visual function of these patients than its length acquired by a single scan.

The IZ was visible in all 158 eyes. Rii et al. reported that the IZ was visible in 43/45 healthy eyes, and fragmentation of the IZ was apparent in the fovea [12]. In our study, however, the IZ was observed as a smooth continuous line in all 158 eyes. The difference might be due to the different OCT systems used in each study. Each radical scan taken by Spectrails in our study comprised 100 averaged scans obtained using eye tracking, while Rii et al. used a different OCT system and probably other technique. In addition, they included some moderate and high myopic eyes (− 8.0 D to + 3.25 D), whereas the eyes in our study had a refraction of − 3 D to + 1 D [12]. However, we found a rather large variation in the IZ area, which ranged from 5.91 to − 57.47 mm^2^. Curcio et al. previously reported that the number of cones varied among healthy subjects [17], and our findings are in agreement with theirs. These results are important for clinical research and for daily clinical practice because ophthalmologists should remember that a small IZ area may be normal. In some patients, the IZ might not be able to continue growing after a certain point. This is not necessarily an indication that the patient is not responding well to the prescribed treatment. Instead, the continuity and smoothness of the IZ might be more informative in clinical settings. But apart from the large range of the IZ area, the 95% confidence interval was 28.06–32.29 mm^2^, which was rather small. As a result, the IZ area might be able to provide some useful information in clinic work. On the other hand, the measurement of area could also serve as a baseline in the follow-up of patients with photoreceptor damage, the change of IZ area might be considered as a sign of change in photoreceptor. Moreover, as the symmetry between the two eyes (*r* = 0.916, *p* <  0.0001), the status or area of the contralateral eye could provide as reference.

The IZ area was also correlated with the volumes of the full retina, ONL, and possibly the ELM-BM and photoreceptors. These correlations were still observed after adjusting for age, gender, and AL. These results suggested that eyes with a large IZ area might contain more photoreceptors, probably cones, in the macula. This is particularly relevant to clinical practice because it was reported that the age-related accumulation of lipofuscin was related to the development of age-related macular degeneration [18], and a photoreceptor density might increase the vulnerability of the eye to such diseases.

In this study, only healthy Chinese subjects were enrolled and only one OCT system was used. Therefore, our findings need to be verified by others, and the possible correlation between IZ area and vision in patients with macular diseases should also be evaluated.

## Conclusion

In our present research, we successfully measured the IZ area in healthy adults using a commercially available OCT system for the first time. The IZ area showed large variability, and eyes with a large IZ area seemed to contain more photoreceptors, probably cones, in the macula.

## Additional file

Additional file 1: **Figure S1.** Schematic diagrams of the mean cone interdigitation zone area and length in 12 directions. The outer ring is 15 ° from the central fovea. IZ = interdigitation zone; I = inferior; INL = inferior nasal lower; INU = inferior nasal upper; ITL = inferior temporal lower, ITU: inferior temporal upper, N = nasal; S = superior; SNL: superior nasal lower; SNU = superior nasal upper; STL = superior temporal lower; STU: superior temporal upper; T = temporal. **Figure S2.** Bland–Altman plots of the repeatability and reproducibility of the IZ area measurements. IZ = interdigitation zone; SD = standard deviation. **Table S1.** Clinical characteristics of the subjects and eyes. **Table S2.** Cone interdigitation area and lengths in 12 directions of the left and right eyes. **Table S3.** Binocular symmetry of the interdigitation zone areas and lengths in 12 directions. (DOCX 1801 kb)
