# Supplementary material for: Area of the cone interdigitation zone in healthy Chinese adults and its correlation with macular volume
Source: BMC Ophthalmol. 2018 Aug 1;18:188. doi: 10.1186/s12886-018-0862-7 (PMC6090954; doi:10.1186/s12886-018-0862-7)
Supplement: Supplementary file 1 — Figure S1. Schematic diagrams of the mean cone interdigitation zone area and length in 12 directions. The outer ring is 15 ° from the central fovea. IZ = interdigitation zone; I = inferior; INL = inferior nasal lower; INU = inferior nasal upper; ITL = inferior temporal lower, ITU: inferior temporal upper, N = nasal; S = superior; SNL: superior nasal lower; SNU = superior nasal upper; STL = superior temporal lower; STU: superior temporal upper; T = temporal. Figure S2. Bland–Altman plots of the repeatability and reproducibility of the IZ area measurements. IZ = interdigitation zone; SD = standard deviation. Table S1. Clinical characteristics of the subjects and eyes. Table S2. Cone interdigitation area and lengths in 12 directions of the left and right eyes. Table S3. Binocular symmetry of the interdigitation zone areas and lengths in 12 directions. (DOCX 1801 kb) [file 12886_2018_862_MOESM1_ESM.docx]

**Supplementary Information**

**
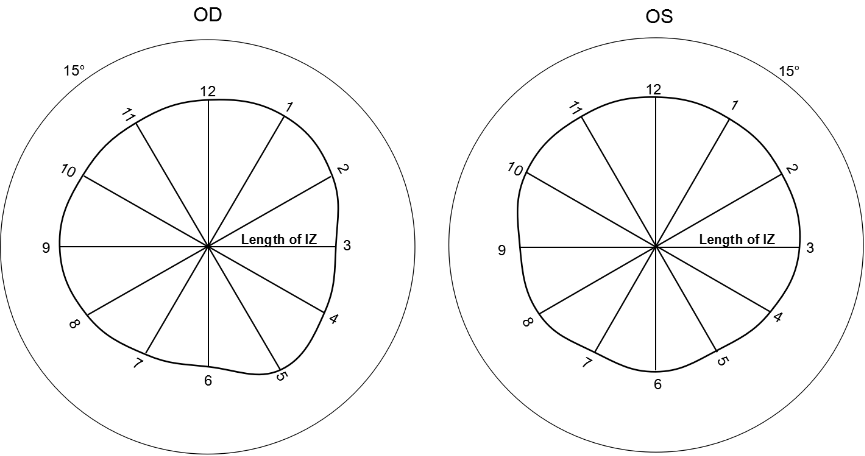
**

**Figure S1.** Schematic diagrams of the mean cone interdigitation zone area and length in 12 directions. The outer ring is 15 ° from the central fovea. IZ = interdigitation zone; I = inferior; INL = inferior nasal lower; INU = inferior nasal upper; ITL = inferior temporal lower, ITU: inferior temporal upper, N = nasal; S = superior; SNL: superior nasal lower; SNU = superior nasal upper; STL = superior temporal lower; STU: superior temporal upper; T = temporal.

**
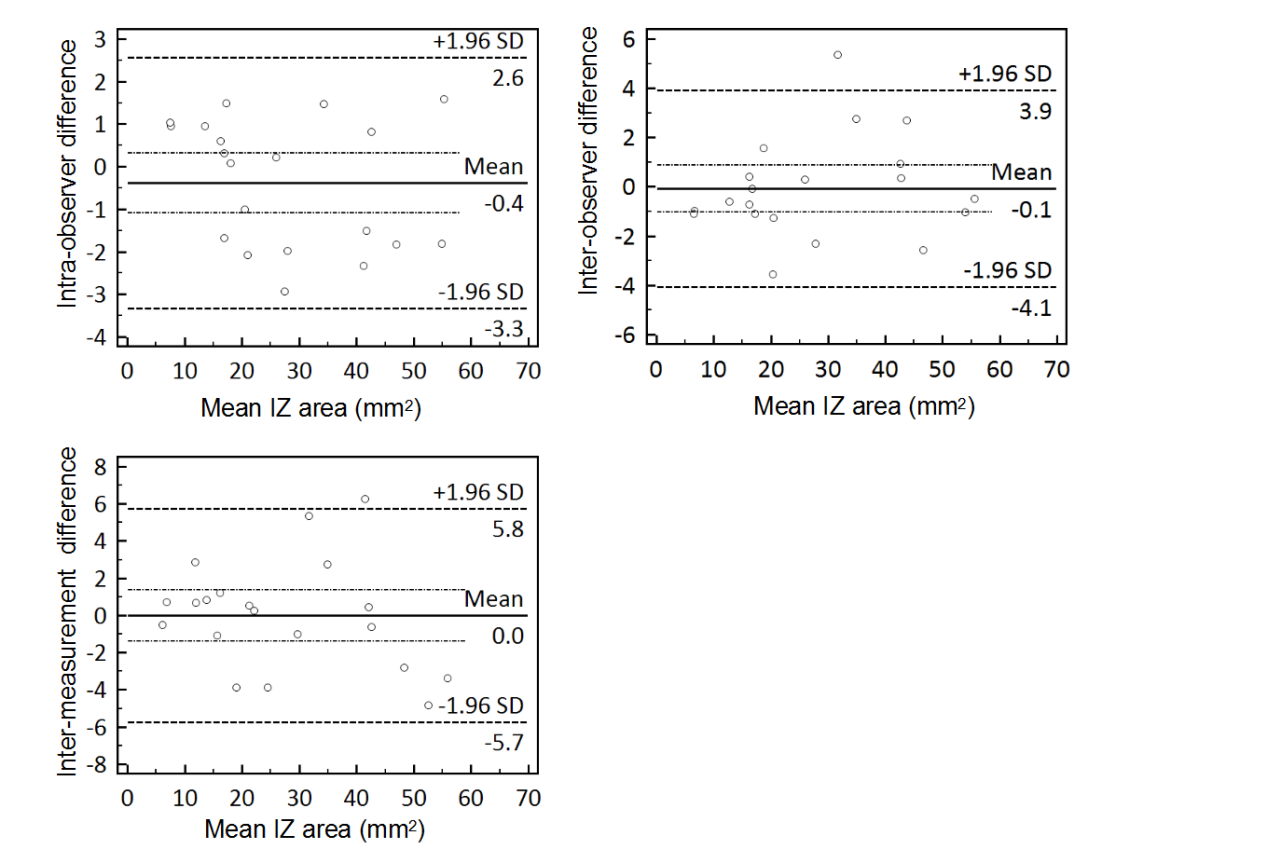
**

**Figure S2.** Bland–Altman plots of the repeatability and reproducibility of the IZ area measurements. IZ = interdigitation zone; SD = standard deviation.

**Table S1** Clinical characteristics of the subjects and eyes

| Characteristic | Value |
| --- | --- |
| Number of eyes | 158 |
| Age (years) | 32.13 ± 12.08 |
| Males | 71 (44.94%) |
| AL (mm) | 30.22 ± 12.70 |
| SE (D) | −0.80 ± 1.04 |
| IOP (mmHg) | 14.41 ± 2.89 |
| BCVA (LogMAR) | 0.01 ± 0.05 |

AL = axial length; BCVA = best-corrected visual acuity; D = diopter; SE = spherical equivalent; IOP = intraocular pressure.

Values are presented as *n* (%) or means ± standard deviation.

**Table S2.** Cone interdigitation area and lengths in 12 directions of the left and right eyes

|  | Right eye | Left eye | *P* value* |
| --- | --- | --- | --- |
| IZ area (mm^2^) | 29.74 ± 13.61 | 29.79 ± 12.39 | 0.981 |
| IZ length (mm) |  |  |  |
| S | 3.27 ± 0.83 | 3.30 ± 0.84 | 0.839 |
| SNU | 3.35 ± 0.82 | 3.26 ± 0.96 | 0.564 |
| SNL | 3.13 ± 0.82 | 3.23 ± 0.86 | 0.443 |
| N | 2.80 ± 0.57 | 2.97 ± 0.58 | 0.072 |
| INU | 2.97 ± 0.73 | 2.91 ± 0.72 | 0.618 |
| INL | 2.75 ± 0.79 | 2.64 ± 0.72 | 0.386 |
| I | 2.70 ± 0.82 | 2.68 ± 0.75 | 0.893 |
| ITL | 2.75 ± 0.83 | 2.68 ± 0.74 | 0.583 |
| ITU | 3.07 ± 0.91 | 2.89 ± 0.77 | 0.184 |
| T | 3.26 ± 0.89 | 3.15 ± 0.74 | 0.402 |
| STL | 3.15 ± 0.96 | 3.16 ± 0.85 | 0.926 |
| STU | 3.19 ± 0.94 | 3.22 ± 0.90 | 0.896 |

I = inferior; INL = inferior nasal lower; INU = inferior nasal upper; ITL = inferior temporal lower, ITU: inferior temporal upper, N = nasal; S = superior; SNL: superior nasal lower; SNU = superior nasal upper; STL = superior temporal lower; STU: superior temporal upper; T = temporal. Values are presented as means ± standard deviation.*Student’s *t* test was used to compare the variables between the right and left eyes.

**Table S3.** Binocular symmetry of the interdigitation zone areas and lengths in 12 directions

|  | Mean difference | Standard deviation | *P* value* |
| --- | --- | --- | --- |
| IZ area (mm^2^) | −0.06 | 5.48 | 0.94 |
| IZ length (mm) |  |  |  |
| S | −0.08 | 0.47 | 0.18 |
| SNU | 0.04 | 0.74 | 0.66 |
| SNL | −0.17 | 0.58 | 0.03* |
| N | −0.17 | 0.53 | 0.02* |
| INU | 0.00 | 0.62 | 0.96 |
| INL | 0.08 | 0.54 | 0.24 |
| I | 0.03 | 0.48 | 0.60 |
| ITL | 0.09 | 0.52 | 0.21 |
| ITU | 0.02 | 0.56 | 0.82 |
| T | −0.07 | 0.54 | 0.34 |
| STL | −0.11 | 0.50 | 0.11 |
| STU | 0.15 | 0.54 | 0.03* |

I = inferior; INL = inferior nasal lower; INU = inferior nasal upper; ITL = inferior temporal lower, ITU: inferior temporal upper, N = nasal; S = superior; SNL: superior nasal lower; SNU = superior nasal upper; STL = superior temporal lower; STU: superior temporal upper; T = temporal.

*Paired *t* tests were used to determine binocular symmetry.
